# The Important Role of Ion Transport System in Cervical Cancer

**DOI:** 10.3390/ijms23010333

**Published:** 2021-12-29

**Authors:** Yih-Fung Chen, Meng-Ru Shen

**Affiliations:** 1 Graduate Institute of Natural Products, College of Pharmacy, Kaohsiung Medical University, Kaohsiung 80708, Taiwan; yihfungchen@kmu.edu.tw; 2 Department of Medical Research, Kaohsiung Medical University Hospital, Kaohsiung 80708, Taiwan; 3 Department of Pharmacology, College of Medicine, National Cheng Kung University, Tainan 70101, Taiwan; 4 Department of Obstetrics and Gynecology, National Cheng Kung University Hospital, College of Medicine, National Cheng Kung University, Tainan 70101, Taiwan

**Keywords:** cervical cancer, cell volume regulation, K^+^-Cl^−^ cotransport, volume-sensitive Cl^−^ channels, store-operated Ca^2+^ entry, stromal interaction molecule (STIM), Orai, migration, cell cycle progression

## Abstract

Cervical cancer is a significant gynecological cancer and causes cancer-related deaths worldwide. Human papillomavirus (HPV) is implicated in the etiology of cervical malignancy. However, much evidence indicates that HPV infection is a necessary but not sufficient cause in cervical carcinogenesis. Therefore, the cellular pathophysiology of cervical cancer is worthy of study. This review summarizes the recent findings concerning the ion transport processes involved in cell volume regulation and intracellular Ca^2+^ homeostasis of epithelial cells and how these transport systems are themselves regulated by the tumor microenvironment. For cell volume regulation, we focused on the volume-sensitive Cl^−^ channels and K^+^-Cl^−^ cotransporter (KCC) family, important regulators for ionic and osmotic homeostasis of epithelial cells. Regarding intracellular Ca^2+^ homeostasis, the Ca^2+^ store sensor STIM molecules and plasma membrane Ca^2+^ channel Orai proteins, the predominant Ca^2+^ entry mechanism in epithelial cells, are discussed. Furthermore, we evaluate the potential of these membrane ion transport systems as diagnostic biomarkers and pharmacological interventions and highlight the challenges.

## 1. Introduction: An Overview of the Epidemiology and Pathogenesis of Cervical Cancer

Cervical cancer is the fourth most frequently diagnosed cancer and the fourth leading cause of cancer death in women, with an estimated 604,000 new cases and 342,000 deaths worldwide in 2020 [1]. Most cervical cancer cases are diagnosed among women in low-income and middle-income countries [1,2,3]. The most common type of cervical cancer is squamous cell carcinoma (SCC), which develops from cervical intraepithelial neoplasia (CIN). The main risk factor for cervical carcinoma is human papillomavirus (HPV) infection, which drives several critical molecular events in cervical cancer development [4]. The integration of the HPV genome into the host chromosome of cervical epithelial cells is a critical early event in the malignant progression of cervical lesions. The HPV oncoproteins E6 and E7 are responsible for the initial pathomolecular changes in cervical epithelial cells. The viral proteins inactivate two main tumor suppressor proteins, p53 and retinoblastoma (Rb). Inactivation of these host proteins disrupts DNA repair machinery and apoptosis, leading to uncontrolled cell proliferation. As a result, multiple genes involved in DNA repair, cell proliferation, growth factor activity, angiogenesis, and mitogenesis become highly expressed in CIN and cervical cancer [5]. This genomic instability allows HPV-infected cells to progress towards invasive carcinoma.

Despite the critical role of oncogenic HPV in cervical dysplasia and cervical cancer development, only a certain percentage of the persistent HPV infections eventually develop into cervical carcinoma, independent of their association with HPVs [1,6]. Much evidence indicates that HPV infection is a necessary but insufficient cause of cervical cancer [1,7,8]. More than 80% of women have been infected with HPV, but only a small proportion of women develop cervical cancer. The high-risk HPV types, mainly HPV16 and HPV18, cause cervical cancer in more than 80% of the cases [1]. Some low-risk HPVs are known to cause benign cervical lesions and genital warts. The incidence of invasive cervical cancer varies considerably in different populations, reflecting the influence of variations in environmental factors, some sexually transmittable infections (HIV and *Chlamydia trachomatis*), primary prevention by HPV vaccination, Pap smear surveillance, and treatments of pre-invasive lesions [1]. Additional factors may contribute to the pathogenesis of cervical cancer [9,10]. Therefore, the cellular pathophysiology of cervical cancer is still worth being investigated.

Membrane ion transport systems are the gatekeepers for cells and organelles, thereby controlling the transportation of various exogenous/endogenous substances via influx/efflux mechanisms. Recent studies have uncovered the remodeling of ion homeostasis during cancer progression and explored the novel functions of membrane ion transport systems in the regulation of tumor malignancy (see reviews [11,12,13,14,15,16,17]). However, little information is available on the roles of membrane ion transport systems in the neoplastic transformation and progression of cervical epithelial cells.

This review focuses on the membrane ion transport processes involved in cell volume regulation and intracellular Ca^2+^ homeostasis of epithelial cells and how these transport systems are themselves regulated by the tumor microenvironment. For cell volume regulation, we focused on the volume-sensitive Cl^−^ channels and K^+^-Cl^−^ cotransporter (KCC) family, important regulators for the ionic and osmotic homeostasis of epithelial cells. Regarding intracellular Ca^2+^ homeostasis, the Ca^2+^ store sensor STIM molecules and plasma membrane Ca^2+^ channel Orai proteins, the predominant Ca^2+^ entry mechanism in epithelial cells, are discussed. Furthermore, we summarized recent progress in studies on the potential for diagnostic biomarkers and pharmacological interventions of these membrane ion transport systems. Finally, the challenges that remain to be further dissected are also discussed.

## 2. Cell Volume Regulation and Volume-Sensitive Cl^−^ Channels

The maintenance of homeostasis is a fundamental cellular property [18]. The regulation of cell volume, one of the fundamental cellular homeostatic mechanisms, is a widespread process that enables cells to maintain their average volume in the face of alternations in extracellular osmolarity [19]. Cells have to avoid drastic cell volume changes, which jeopardize structural integrity and constancy of the intracellular environment. Even under the constant extracellular osmolarity, cell volume is frequently challenged by the membrane transport of osmotically active substances and the formation consumption of cellular osmolytes by metabolism [20]. Accumulating evidence supports that cell volume homeostasis does not simply mean volume constancy but also serves as the integration of events in regulating cellular mechanic properties and functions, including epithelial transport, metabolism, cell proliferation, differentiation, migration, and cell death [21,22,23,24,25]. Moreover, cancer cell migration and invasion that are committed steps in tumor metastasis also involve extensive modification of the cell volume and geometrical morphology at different regions of the cells [26,27].

The homeostasis of cell volume involves the continuous functioning of the ion transport process across the plasma membrane and the fluxes of organic osmolytes and metabolites [20,28,29]. In response to the hypotonic stress, cells defend themselves by activating an efflux of cell osmolytes, including ions and specific organic molecules together with osmotically responsive water, to accomplish the process of regulatory volume decrease (RVD) [28,30]. Different ion transport systems have been reported to be responsible for the loss of K^+^ and Cl^−^ in response to cell swelling [31,32]. In most cell types, the predominant pathway for RVD-associated loss of K^+^ and Cl^−^ is the selective activation of separate volume-sensitive K^+^ and Cl^−^ channels [19,33,34]. Several proteins have been investigated and discussed as the channel mediating the release of Cl^–^ during RVD, e.g., ICln [35] and SWELL1 (also known as the leucine-rich repeat-containing protein 8A, LRRC8A) [36]. Another important pathway for RVD is the K^+^-Cl^−^ cotransporter (KCC), which transports K^+^ and Cl^−^ stoichiometrically in either direction across plasma membranes and is observed predominantly in erythrocytes [37,38,39], neurons [40], and some epithelial cells [41].

**Figure 1:**
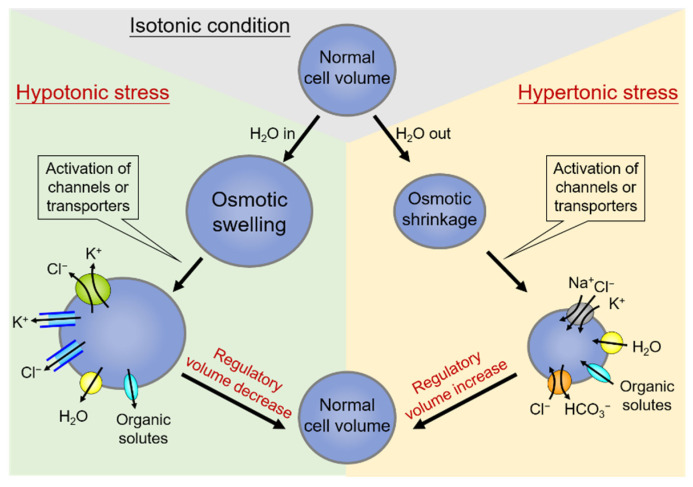
Regulation and homeostasis of cell volume: regulatory volume decrease (RVD) and regulatory volume increase (RVI). Under hypotonic stress conditions of cell swelling (**left side**), the cell activates the regulatory volume decrease (RVD). In this condition, the volume-regulatory accumulation and loss of electrolytes are mediated by the activity of membrane channels or transporters responsible for the loss of K^+^ and Cl^−^, and organic solutes along with water in response to cell swelling. On the contrary, cell volume shrinks due to water extrusion under hypertonic stress (**right side**), and a counter-response of regulatory volume increase (RVI) occurs to restore normal cell volume. Shrunken cells can thereby increase their volume towards the original levels by upregulating the net influx of cell osmolytes, including Na^+^, Cl^−^, and often K^+^, and concurrent uptake of water.

On the other hand, hypertonic stress that causes the osmotic shrinkage of cells can activate the regulatory volume increase (RVI). Shrunken cells can thereby increase their volume towards the original levels by upregulating the net influx of cell osmolytes, including Na^+^, Cl^−^, and often K^+^ as well, and concurrent uptake of water [42]. The central ion transport systems accomplishing electrolyte accumulation in shrunken cells are the Na^+^-K^+^-2Cl^−^ cotransporter (NKCC) and the Na^+^/H^+^ exchanger (NHE) [43,44]. The effects of NHE also lead to the alkalization of the cell and thus the coincidental activation of the Cl^−^/HCO_3_^−^ exchanger [45]. A schematic diagram summarizing the regulation and homeostasis of cell volume by the process of RVD and RVI is shown in Figure 1.

### 2.1. Volume-Sensitive Cl^−^ Channels Associated with Human Cervical Carcinogenesis

Volume-regulated anion channel (VRAC) is ubiquitously expressed in vertebrate cells [46,47]. In addition to volume regulation, VRACs play essential roles in several critical physiological processes, such as osmolyte transport, metabolism, hormone release, cell migration, proliferation, and differentiation [32,48,49]. Among different VARCs, the activation of volume-sensitive Cl^−^ channels has been demonstrated essential in the volume regulation of several non-excitable and excitable cell types [29,50]. Volume-sensitive Cl^−^ channels have been reported to participate in cell survival and migration, given their ability to coordinate ion and water movement through the plasma membrane [19,51]. However, the expression and functional significance of volume-sensitive Cl^−^ channels in cervical carcinoma have been less studied.

Our research group was the first to study the roles of the preferentially activated Cl^−^ channel in cervical carcinoma [52,53]. The activations of volume-sensitive Cl^−^ currents in various human cervical epithelial cells representing different stages of cervical carcinogenesis were investigated using the whole-cell patch-clamp technique. It was found that hypotonicity activated an outward rectified, ATP-dependent, volume-sensitive Cl^−^ current in human cervical cancer cells, including four cervical cancer cell lines, primary cells of carcinoma in situ, and invasive cancer of the cervix, but not in non-cancerous HPV-immortalized cells and normal cervical epithelial cells. This was the first report that suggested that the activation of volume-sensitive Cl^−^ channels is associated with malignant transformation of human cervical squamous epithelium independent of various cancer stages, histopathological types, and HPV DNA positivity. Moreover, the cAMP-mediated Cl^−^ currents were ubiquitously activated in all cervical squamous cells studied, regardless of the stages of carcinogenesis, indicating that not all of the Cl^−^ channels are uniformly upregulated during cervical carcinogenesis [52].

In addition to the osmotic challenge, all cells possess mechanisms to maintain the homeostasis in cell volume precisely during cell cycle progression [54,55,56,57]. Especially at the G1/S transition, cells undergo a significant increase in size, disturbing the homeostasis of cell volume. Thus the process of RVD is activated to balance such cell volume decrease. It has been shown that the differential expression of K^+^ channels and accompanying membrane potential changes are the key to cell cycle checkpoints [58,59]. However, whether the progression of the cell cycle is accompanied by differential expression of VRAC activity was less studied. We have previously demonstrated that cell cycle progression correlates with the expression of VRAC activity by employing the whole-cell patch-clamp recording in human cervical cancer cells under various characteristics of the cell cycle conditions [60]. The arrest of cell growth in the G0/G1 phase by aphidicolin was accompanied by a significant decrease in the VRAC current density. However, the inhibited VRAC activity was recovered by the re-entry into the cell cycle upon aphidicolin removal.

Moreover, pharmacological blockade of VRACs caused proliferating cervical cancer cells to arrest in the G0/G1 stage, indicating that activity of VRAC is critical for G1/S checkpoint progression. This was the first study that provided important information on the functional significance of VRACs in the cell cycle clock of human cervical cancer cells. These results, together with reports showing the inhibition of cell proliferation by blockage of volume-regulatory K^+^ and Cl^−^ channels in other cell types, such as human peripheral T lymphocytes [61], endothelial cells [62], and microglial cells [63], indicate a possible role for these volume-sensitive channels in mitogenesis.

### 2.2. Differential Osmosensing Signaling Pathways of Volume-Sensitive Cl^−^ Channels Associated with Human Cervical Carcinogenesis

The volume-sensitive Cl^−^ channels, leading to RVD, were distinctly activated in cervical cancer cells with different tumor potentials [52,53,60,64]. One would be curious whether the osmosensing signalings involved in mediating RVD and controlling activities of volume-sensitive Cl^−^ channels are also altered in different cervical cell types. Several signaling molecules have been suggested as potential mediators of RVD, including intracellular Ca^2+^, calmodulin-dependent protein kinase, protein kinase C (PKC), cyclic adenosine monophosphate (cAMP), and protein kinase A (PKA) [32,65,66,67,68]. The pharmacological screening on the possible signaling pathways involved in cell volume regulation reported that the signaling pathways mediating RVD in different cervical cell types involve the differential activation of distinct PKC isoforms [68]. Phospholipase C (MAPK) signaling with downstream activation of conventional, classic PKCs was involved in the RVD response of cervical cancer cells. On the other hand, different PKC isoforms unrelated to upstream PLC regulation were involved in the RVD of HPV-immortalized and normal cervical epithelia. Results from the whole-cell patch-clamp studies and immunofluorescence staining suggested the involvement of the conventional PKC-α, but not PKC-β or PKC-γ, in the regulation of RVD responses and activation of volume-sensitive Cl^−^ channels in cervical cancer cells [69]. Even though the vast amount of studies demonstrated that the signaling of PKCs regulates multiple pathways relevant for cell cycle progression, tumorigenesis, and metastasis, the relevance of individual PKC isoforms in the progression of human cancer is still a matter of controversy [70]. The above-mentioned results, together with the previous studies showing the differential RVD responses in cervical cells with different malignant potentials [52,53,60], suggested the differential role of individual PKCs in cervical carcinogenesis involves the differential regulatory mechanisms on cell volume regulation and volume-sensitive Cl^−^ channels.

The cytoskeleton is a dynamic intracellular structure that plays an essential role in regulating most biological processes, including the stability of cell shape, the onset of cell movement, and wound healing. The dynamic rearrangement of the cytoskeleton has also been shown involved in cell volume regulation in response to osmotic challenges [71,72,73]. In RVD, actin filaments are depolymerized during cell swelling, followed by actin polymerization at the phase of volume recovery. Cell swelling also increases microtubule stability and stimulates the expression of tubulin. The differential roles of actin filaments and microtubules in regulating volume-sensitive Cl^−^ channels and RVD responses in human cervical cancer were investigated with the model of HT-3 cells [74]. The results from whole-cell voltage clamping and cell size monitoring showed that the drugs that affect cytoskeleton integrity have variable effects on the expression of volume-sensitive Cl^−^ currents of cervical cancer cells. Depolymerization of actin with cytochalasin B potentiated the expression of Cl^−^ currents in hypotonic stress and significantly prolonged RVD responses. In contrast, stabilization of actin polymerization by phalloidin abolished the increase in hypotonicity-elicited whole-cell Cl^−^ conductance and retarded the cell volume recovery. Inhibition of microtubule assembly by colchicine had no effects on volume-sensitive Cl^−^ current and RVD responses. Stabilization of microtubule by paclitaxel dose-dependently inhibited the activation of volume-sensitive Cl^−^ channels and the process of RVD. These point to the importance of the functional integrity of actin filaments and microtubules in maintaining the effective RVD responses and activation of volume-sensitive Cl^−^ channels in human cervical cancer cells.

However, it should be noted that the architecture of actin filaments seems to have varying effects on RVD, and volume-sensitive Cl^−^ channels in different cell types be diverse in a cell-dependent manner, indicating the cell-type-specific requirement of actin cytoskeleton for cell-volume regulation. Some have similar findings to ours [74], showing that disruption of actin filaments potentiates the activation rate of volume-sensitive Cl^−^ channels [75,76,77], whereas some other reports indicated that actin polymerization is required for the activation of volume-sensitive Cl^−^ channels [78,79,80]. This also implies that different channels and transporters involved in volume regulation may have various associations with the cytoskeleton.

The above-mentioned studies on the association and osmosensing signaling of volume-sensitive Cl^−^ channels in human cervical epithelial cells may provide a model for a better understanding of the molecular carcinogenesis of human cervical cancer. In addition, volume-sensitive Cl^−^ channels in cervical cancer cells may offer a potential target for therapeutic intervention of cervical carcinoma and the reversal of malignant progression in human cervical carcinogenesis [81].

## 3. K^+^-Cl^−^ Cotransport and K^+^-Cl^−^ Cotransporter (KCC) Family

The electroneutral K^+^-Cl^−^ cotransport in either direction across the plasma membrane is another important pathway for RVD [40]. K^+^-Cl^−^ cotransport was first characterized as a hypotonically activated, Cl^−^-driven, K^+^ efflux mechanism in human red blood cells (RBCs) responsible for mediating RVD and maintaining cell volume in response to hypotonic cell swelling [82,83]. K^+^-Cl^−^ cotransport is one of the major K^+^ and Cl^−^ flux pathways in erythrocytes, neurons, and epithelial cells [38,39,40]. The activity of KCC plays a significant role in ionic and osmotic homeostasis, cell volume regulation, and transepithelial ion transport [41].

Several physiological changes are known to stimulate the activity of K^+^-Cl^−^ cotransport, such as cell swelling, decreased intracellular pH, increased partial pressure of oxygen, and urea accumulation [84]. Some pharmacological activators of K^+^-Cl^−^ cotransport have also been identified, including the oxidizing reagents (e.g., hydroxylamine, H_2_O_2_, and NO) and the thiol-alkylating reagent N-ethylmaleimide (NEM) [85]. The intracellular signaling cascades comprising multiple protein phosphorylation and dephosphorylation also play an important role in modulating K^+^-Cl^−^ cotransport activity [86,87,88,89,90,91,92,93]. K^+^-Cl^−^ cotransport is stimulated by kinase inhibition (e.g., thiol reagents, staurosporine, and genistein) and inactivated by phosphatase inhibition (e.g., okadaic acid and calyculin) [84]. Protein phosphatases PP1A- and PP2A-dependent active dephosphorylation and the With-No-Lysine (K) (WNK)–STE20-Proline Alanine rich Kinase (SPAK)/Oxidative Stress Responsive Kinase 1 (OSR1) cascade-mediated inhibitory phosphorylation are implicated in modulating the activity of KCC isoforms, respectively [94,95,96,97].

The electroneutral K^+^-Cl^−^ cotransport is carried out by the four distinct members of K^+^-Cl^−^ cotransporter (KCC) encoded by the *Solute Carrier 12* (*SLC12*) gene family of electroneutral cation-chloride cotransporters [98,99], namely KCC1 (*SLC12A4*) [100], KCC2 (*SLC12A5*) [101,102], KCC3 (*SLC12A6*) [103,104], and KCC4 (*SLC12A7*) [104]. The four KCCs were predicted to share conserved structural features [105,106], including a central core of 12 hydrophobic transmembrane helices (TMs) flanked by the intracellular hydrophilic N- and C-terminal ends and a large extracellular domain with several N-linked glycosylation sites between the 5th and 6th transmembrane helixes (TMs). The intracellular C-terminal domain contains important sites for phosphorylation and dephosphorylation that regulate the expression, trafficking, and activity of KCCs [86,87,88,89,90,91,92,93,107]. With the recent advances in cryo-electron microscopy (cryo-EM), the structures of KCC isoforms have also been resolved [107,108,109,110,111], which revealed new chemical and biological insights into the structural topology of KCCs. The cryo-EM structural data confirmed an ordered and glycosylated loop in the extracellular domain between TM5 and TM6 and two intracellular domains with numerous phosphorylation sites at the N- and C-terminus. Results of cryo-EM structures indicated the dimeric organization of KCCs through the dimerization interphase formed by TM11 and TM12, except mouse KCC4 as a monomer [110].

The activities of KCC1, KCC3, and KCC4, so-called classical volume-regulated or swelling-activated KCCs, are osmotically sensitive and participate in cell volume regulation [112,113,114]. The neuronal-specific isoform KCC2 is also activated by cell swelling and exhibits the constitutive K^+^-Cl^−^ cotransport activity under isotonic conditions [102]. Therefore, KCC2 is vital in maintaining intra-neuronal Cl^−^ concentration and is required to establish GABAergic hyperpolarizing synaptic inhibition [40,115]. Indeed, mutations in the *SLC12A5* gene have been found in subjects affected by brain disorders, particularly epilepsy [115,116,117].

KCC1, ubiquitously detected in mammalian cells and tissues, is considered to act as a “housekeeping” KCC isoform for cell volume regulation and transepithelial ion transport [112]. No specific human disorders have been linked to mutations in the *SLC12A4* gene, and K^+^-Cl^−^ cotransport activity in RBCs is undiminished in *KCC1* knockout mice. This suggested the evolutionary redundancy of KCCs and other unknown functions of KCC1.

Loss-of-function mutations in the human *KCC3* gene cause an autosomal disease, known as Andermann syndrome or the agenesis of the corpus callosum with peripheral neuropathy (ACCPN) [118]. The symptoms of Andermann syndrome include various degrees of sensorimotor neuropathy, mental disability, psychotic symptoms, and complete or partial ACCPN. *KCC3* KO mice display slowly progressive deafness, peripheral neurodegeneration, reduced seizure threshold, neurogenic hypertension, and locomotor dysfunction [119,120,121], which are consistent with certain features of ACCPN. Moreover, KCC3 plays a vital role in regulating cell proliferation [122].

KCC4, predominantly found in the heart and kidney, also contributes to volume regulation [114]. Recently. A novel de novo deletion in the *KCC4* gene was identified to be associated with the sporadic congenital hydrocephalus [123]. Mice with *KCC4* gene disruption develop progressive deafness and renal tubular acidosis [124].

The details of structural insights, molecular characterization, physiological functions, and pathological defects of KCC isoforms have been comprehensively reviewed [102,112,113,114,115,125]. Here we focused on the emerging importance of K^+^-Cl^−^ cotransport and individual KCC isoforms in cervical epithelial carcinogenesis and tumor malignant behaviors.

### 3.1. K^+^-Cl^−^ Cotransport Activity Affects Malignant Transformation, Proliferation, and Invasion of Cervical Epithelial Cells

Our long-term and systemic research highlighted the emerging importance of K^+^-Cl^−^ cotransport in cervical epithelial carcinogenesis and tumor malignancies. Firstly, whether cervical malignancy is accompanied by differential expression of volume-sensitive KCCs was investigated [126]. The K^+^-Cl^−^ cotransport activity of normal human cervical epithelial cells was quiescent in normal physiological conditions but did not respond to hypotonic stress. In contrast, cervical cancer cells have K^+^-Cl^−^ cotransport activity which was also nearly quiescent in normal physiological conditions, but high transport rates were observed in response to the hypotonic challenge. Results of reverse transcription polymerase chain reaction (RT-PCR) indicated that cervical carcinogenesis is accompanied by the up-regulation of mRNA transcripts in volume-sensitive KCC1, KCC3, and KCC4. Moreover, K^+^-Cl^−^ cotransport activities were downregulated by protein phosphatase inhibitors and upregulated by protein kinase inhibitors, indicating that a phosphorylation cascade modulates the volume-sensitive KCC in cervical cancer cells. Evidence from molecular identification and functional flux studies demonstrated that the malignant transformation of cervical epithelial cells is associated with the differential activity and expression of volume-sensitive KCC isoforms, which plays a significant role in the volume regulation of cervical cancer cells [126].

Further study demonstrated the critical role of K^+^-Cl^−^ cotransport as an important modulator of growth and invasiveness in human cervical cancer [127]. In carcinogenesis, cervical epithelial cells breach the basement membrane to proliferate and migrate within the adjacent connective tissue. An important event in the dissolution of the basement membrane matrix involves the activation of the matrix metalloproteinases (MMPs) cascade, which is accompanied by the altered expression of several cell adhesion molecules in the transformed cells [128,129,130]. Cervical cancer cells expressing a dominant-negative loss-of-function KCC mutant exhibited inhibited cell growth accompanied by decreased expressions of the cell cycle regulators retinoblastoma and cdc2 kinase, as well as the inhibited cellular invasiveness accompanied by reduced expression of α_v_β_3_ and α_6_β_4_ integrins and reduced activities of MMPs [127]. Inhibited tumor growth of the subcutaneous implanted loss-of-function KCC mutant expressing cervical cancer cells in severe combined immunodeficient (SCID) mice confirmed the crucial role of KCC in promoting cervical cancer growth and invasion [127]. Thus, blockade of K^+^-Cl^−^ cotransport may be a useful adjunctive therapeutic strategy to retard or prevent cervical cancer invasion.

The metastatic and invasive properties of cancer cells are stimulated by specific growth factors, which poses severe problems to successful cancer treatment. For example, the insulin-like growth factor 1 (IGF-1) system performs multiple functions in the pathogenesis of different types of cancer [131,132,133]. A further study established the critical role of K^+^-Cl^−^ cotransport in IGF-1 signaling to promote the growth and spread of cervical cancers [134]. The findings demonstrated that IGF-1 increased KCC expression and activity in parallel with the enhancement of RVD. IGF-1-stimulated biosynthesis of KCC involved the Erk1/2 mitogen-activated protein kinase (MAPK) and phosphatidylinositol 3-kinase signaling pathways. Pharmacological inhibition and genetic modification of K^+^-Cl^−^ cotransport demonstrated that KCC is necessary for IGF-1-induced cancer cell invasiveness and proliferation. These results suggested IGF-1 promotes cervical cancer development and progression in part through its action on KCC [134].

### 3.2. The Distinct Roles of KCC Isoforms in Cervical Carcinogenesis

The emerging importance of individual KCC isoforms in cervical carcinogenesis was enlightened by the investigations in breast cancer [135]. IGF-1 upregulates the activity and expression of KCC3 and KCC4, which are differentially required for IGF-1 receptor signaling to promote the proliferation and invasiveness of breast cancer cells [135]. Further studies substantiated the distinct roles of KCC3 and KCC4 in promoting the conversion of epithelial cells to mesenchymal cells [epithelial-mesenchymal transition (EMT)] and invasiveness of cervical cancer, respectively [136,137].

A key event for cancer invasion and metastasis is EMT. It is considered a potential target for the successful treatment of cancer (as reviewed in [138,139,140]). EMT is known to enhance some cellular functions of tumors, such as increased invasive migration and resistance to anoikis. EMT has been regarded as a hallmark for cancer metastasis in several types of cancer, including breast cancer, cervical cancer, colorectal cancer, and lung cancer. The downregulation of epithelial markers, such as E-cadherin and β-catenin, and upregulation of the mesenchymal markers, such as vimentin and fibronectin, are used to detect the events of EMT.

The literature on the important role of EMT in cervical carcinogenesis is scarce, and little information is available on the roles of membrane ion transport processes in the regulation of EMT to promote cervical cancer progression [141]. We were the first group to investigate if KCC activity is involved in the regulation of EMT by the model of cervical carcinoma [136]. The blockade of KCC activity increased the colocalization of E-cadherin and β-catenin in cervical cancer SiHa and CaSki cells [136]. Moreover, KCC3-overexpressed cervical cancer cells, but not KCC1- or KCC4-overexpressed ones, displayed downregulated E-cadherin/β-catenin complex formation by inhibiting transcription of *E-cadherin* gene and accelerating the proteosome-dependent degradation of β-catenin protein [136]. The promoter activity assays of various regulatory sequences confirmed that KCC3 expression is a potent negative regulator for *E-cadherin* gene expression. That, therefore, promotes the EMT of cervical cancer cells, thereby stimulating tumor progression.

Another study stresses the critical role of KCC4 in the malignant behaviors of cervical cancer cells, especially IGF-1 and epidermal growth factor (EGF) dependent cancer cell invasiveness [137]. IGF-1 and EGF are known to be overexpressed in most types of cancer tissues and contribute to cancer resistance to existing treatments [131,132,133,142,143,144]. A previous study has shown that IGF-1 and EGF are the two most potent stimulators for gynecologic cancer cell invasiveness [145]. KCC4-specific siRNA significantly attenuated endogenous cellular invasiveness of cancer cells, and the residual cellular invasiveness was much less sensitive to IGF-1 or EGF stimulation. IGF-1 and EGF were shown to stimulate the recruitment of KCC4 from a presumably inactive cytoplasmic pool of endoplasmic reticulum (ER) and Golgi to the front-end plasma membrane of migrating cervical cancer cells. Throughout the process of KCC4 trafficking along the track of actin cytoskeleton, the membrane microdomain of lipid rafts functions as a platform for the association between KCC4 the actin-dependent motor myosin Va. Moreover, KCC4 serves as a membrane scaffold for assembly signal complexes via the association with the actin-binding protein, ezrin, at the lamellipodia of migratory cervical cancer cells. IGF-1-induced membrane trafficking of KCC4 and the interaction between KCC4 and ezrin near the cell surface were dramatically suppressed by the interference with KCC activity by either a pharmacological inhibitor or a dominant-negative loss-of-function mutant. Thus, blockade of KCC4 trafficking and surface expression may provide a potential target for preventing IGF-1- or EGF-dependent cervical cancer metastasis [137].

The novel role of KCC2, known as the neuronal-specific KCC, has also been reported by using the cell model of cervical cells, showing that KCC2 promotes cervical cancer cell migration and invasion by an ion transport-independent mechanism [146]. KCC2 was found widely expressed in several human cancer cell lines, including the cervical cancer cell lines SiHa and Hela. Overexpression of KCC2 in cervical cancer cells enhanced IGF-1-stimulated cell migration and invasiveness. Immunofluorescent analyses suggested that KCC2 increases cervical tumorigenesis via the modulation of cell spreading, actin stress fiber formation, and focal adhesion formation. The novel finding on the role of KCC2 in cervical cancer cells supported that KCC2 expression and function are not restricted to neurons [147].

**Figure 2:**
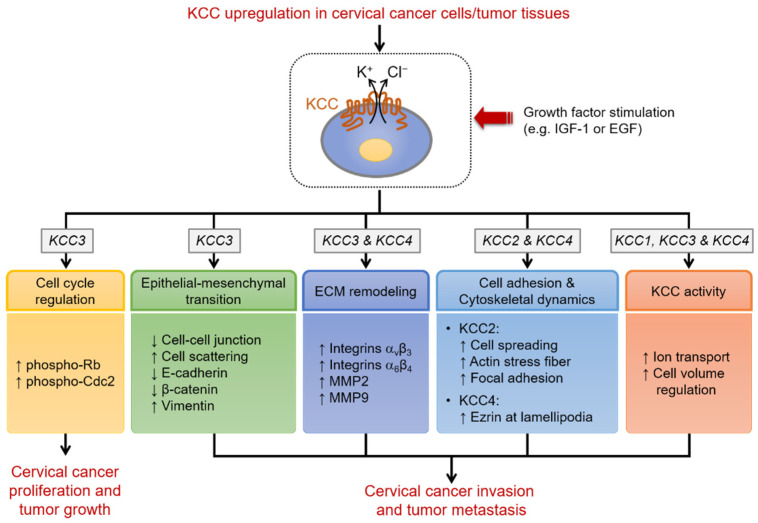
Schematic diagram illustrating the mechanisms by which KCC affects cervical cancer proliferation and invasion. The protumorigenic growth factors IGF-1 and EGF are known potent stimulators for the expression and activity of KCC in cervical cancer cells. K^+^-Cl^−^ cotransport activity plays an important role in controlling cancer cell proliferation via the modulation of phosphorylation two key cell cycle regulators, retinoblastoma (Rb) and cdc2 kinase. Among different KCC isoforms, KCC3 plays a vital role in regulating cell proliferation. The abundance of E-cadherin and β-catenin are affected by the expression and activity of KCC3. Thus, the dissociation of the E-cadherin/β-catenin complex leads to the disorganization of cell-cell junctions, thereby resulting in cervical cancer invasion. On the other hand, the lamellipodia-localized KCC4 functions as a plasma membrane scaffold to regulate cytoskeletal reorganization through the interaction with actin-binding protein, ezrin. The potent effects of KCC4 overexpression on cancer invasion and metastasis also involve the modulation of MMP-2 activity and cell volume control. KCC2 promotes cervical cancer cell migration and invasion by an ion transport-independent mechanism of cell spreading, actin stress fiber formation, and focal adhesion formation.↑, upregulation by KCC; ↓, downregulation by KCC.

The mechanisms by which K^+^-Cl^−^ cotransport activity and individual KCC isoforms affect cervical cancer proliferation and invasion are summarized in Figure 2.

### 3.3. Clinical Implications and Therapeutic Significance of KCC in Cervical Carcinogenesis

Despite the advances in the diagnostic and therapeutic modalities for treating cancer patients, tumor metastasis and cancer recurrence still represent the primary cause of cancer death. Molecules that are consistently upregulated in metastatic and recurrent cancers have the potentials as a reliable biomarker to predict the occurrence, progression, or prognosis of human cancers.

A series of studies have highlighted the critical role of the KCC family in tumor development and progression, suggesting their potentials as the biomarker for the prognosis of cancer patients. Indeed, the studies on cervical cancers have demonstrated the close correlations between the expression of individual KCC isoforms and the clinical outcomes of cancer patients. Immunofluorescent analyses of different KCC isoforms and real-time RT-PCR of laser microdissected tissues suggested that KCC3 is highly expressed in cervical carcinoma whereas KCC4 in metastatic cervical cancer tissues [136,137]. Normal squamous epithelial and non-cancerous stromal tissue express little KCC3 protein, whereas cervical carcinoma and the tumor nest invaded deeply into stromal tissues express abundant KCC3 proteins [136]. Moreover, levels of KCC3 mRNA transcripts expressed in tumor tissues are closely associated with the tumor size. Similar to the expression pattern of KCC3, KCC4 protein is insignificant in non-cancerous cervical squamous epithelial tissues but is obviously expressed at the primary cancerous tissues of the cervix [137]. Higher tumoral expression of KCC4 correlates with poor clinical outcomes, including the more significant percentage of parametrial invasion and pelvic lymph node metastasis, as well as the increased risk of cancer relapse.

The expressions of KCCs and its potent stimulator IGF-1 are abundant in cervical cancer tissues but are nearly undetected in the adjacent normal cervical epithelial and non-cancerous stromal tissues [134]. In addition, IGF-1 and KCC colocalize in the surgical specimens of cervical cancer [134], suggesting the possible autocrine or paracrine effect of IGF-1 on KCC production in vivo. Moreover, the expression of IGF-1 and KCC in surgical specimens shows an excellent linear correlation to tumor size [134], the in vivo indicator of tumor progression. These findings indicated that KCC activation by IGF-1 plays an important role in IGF-1 signaling to promote the growth and spread of cervical cancers. Additionally, KCC4 and its stimulators, EGF and IGF-1, are colocalized in the metastatic cancer tissues [137], suggesting the cooperation between KCC4 and EGF or IGF-1 in tumor metastasis. The results indicated that metastatic cervical cancer tissues express abundant KCC4, which benefits cancer cells in invasiveness. The above-mentioned evidence based on the molecular investigation in surgical specimens of cervical cancer suggested that the clinical outcome of cancer patients is highly associated with the expression of KCC3, KCC4, and their potent stimulators, IGF-1 and EGF [134,135,137]. Therefore, KCC3, KCC4, EGF, and IGF-1 may be a panel of promising diagnostic biomarkers to predict cancer patient outcomes.

Despite several inhibitors or activators that have been shown to manipulate KCC activities effectively, most of them are non-selective for specific KCC isoforms. None of them are utilized as approved therapeutic drugs for cancer treatment [17,148,149]. The discovery and characterization of WNK–SPAK/OSR1 cascade-mediated inhibitory phosphorylation has shed light on the druggable “switch” for the pharmacological manipulation of KCC activity. Furthermore, an improved understanding of WNK/SPAK-mediated KCC activity in cancer cells could reveal novel avenues for therapeutic approaches. Indeed, a previous study has shown that SPAK plays an important role in regulating KCC3-mediated cervical cancer aggressiveness [150]. Mechanistic investigations revealed that KCC3 overexpression led to the increased MMP2 expression and augmented binding of NF-κB to its putative SPAK promoter binding site. This suggested that SPAK promotes KCC3-mediated cervical cancer aggressiveness via the NF-κB/p38 MAPK/MMP2 axis. Thus, the KCC3/SPAK-mediated pathways may be an attractive target for pharmacological intervention of cervical cancer [150]. More importantly, the cryo-EM structures of KCCs have advanced our understanding of the structural topology of KCCs. Further elucidating the structure-activity relationships (SAR) of KCCs will lead to a better understanding of the emerging role of KCCs in carcinogenesis and stimulate structure-based drug discovery of potent and selective modulators of specific KCC isoforms for effective treatment of cancers.

## 4. Intracellular Ca^2+^ Homeostasis and Store-Operated Ca^2+^ Entry (SOCE)

The cytosolic Ca^2+^ is a crucial second messenger involved in controlling diverse cellular functions, such as proliferation, differentiation, survival, migration, and gene expressions [151,152,153]. The increase in the cytosolic Ca^2+^ levels is mainly contributed by the Ca^2+^ fluxes from the extracellular space or the internal Ca^2+^ storage. Store-operated Ca^2+^ entry (SOCE), which constitutes the release of Ca^2+^ from the ER and the influx of Ca^2+^ through the plasmalemmal store-operated Ca^2+^ (SOC) channel, is the primary pathway to increase the cytosolic Ca^2+^ levels in non-excitable cells [154,155]. The molecular determinants underlying the activation of SOCE comprise two families of proteins, the ER Ca^2+^sensors, stromal interaction molecule 1 (STIM1) and STIM2, and the pore-forming proteins of the SOC channel, Orai1 to Orai3 [154,156,157]. STIM proteins are the ER-resident transmembrane protein with several functional domains and protein-protein interaction motifs essential for SOCE activation (see reviews in [158,159]). Once ER Ca^2+^ is depleted, STIM proteins aggregate into oligomers that translocate toward the plasma membrane junctions to interact with and activate Orai proteins, allowing the Ca^2+^ entry. STIM molecules were identified as the microtubule-interacting protein via the direction with the microtubule-plus-end-tracking proteins EB1 and EB3 [160,161]. Several studies have demonstrated the essential roles of microtubules and microtubule-plus-end-tracking mechanisms in the translocation of STIM1 toward the ER-plasma membrane junctions and the following SOCE activation [162,163,164]. With the use of the direct stochastic optical reconstruction microscopy (dSTORM), our recent study provided the ultrastructural view into the activation, aggregation, and translocation of STIM1, as well as the interaction between STIM1, microtubules, and EBs during the dynamic process of SOCE of cervical cancer cells [165]. Upon ER Ca^2+^ depletion, the activated STIM1 interacted with EB1 regardless of undergoing aggregation. Moreover, EB1 silencing did not impair aggregation, but the trafficking of STIM1 to the ER-plasma membrane; and EB3 compensates for the crosstalk between STIM1 and microtubule after EB1-silencing. Results from dSTORM imaging provided novel insights into STIM1 trafficking that is independent of the aggregated state and revealed the role of the microtubule network, end-binding protein EB1, and EB3 in SOCE [165].

The details of structural insights, molecular characterization, physiological functions, pathological defects of STIM and Orai proteins, as well as their dynamic protein-protein interactions that mediated the mediate the activation of SOCE, have been extensively investigated and comprehensively reviewed [166,167,168,169,170,171,172,173,174,175,176]. Increasing evidence demonstrating the essential roles of STIM and Orai proteins have made them potential prognostic biomarkers or antitumor therapeutic targets [177,178,179,180,181,182,183]. Here we updated the recent advances on the importance of STIM/Orai-dependent SOCE in cervical epithelial carcinogenesis and tumor malignant behaviors and the emerging development of SOCE mechanisms as the selective therapeutic target in cervical cancer.

### 4.1. SOCE-Dependent Ca^2^^+^ Signaling Network in Cervical Carcinogenesis

#### 4.1.1. Proliferation and Cell Cycle Regulation

The functional significance of STIM-mediated SOCE in cervical cancer cell proliferation was extensively studied. Investigations in human cervical cancer cells showed that cell proliferation and cell cycle progression were significantly slowed down by STIM1 silencing that was attributed to the increased expression of cyclin-dependent kinase inhibitor p21 protein and decreased levels of phosphatase Cdc25C protein [184]. Results from the intracellular Ca^2+^ measurement in cervical cancer cells synchronized in different cell cycle status found the fluctuating SOCE activity during cell cycle progression, in which SOCE is upregulated in G1/S transition and downregulated from S to G2/M transition [185]. Mechanistic investigations showed that the blockade of SOCE activity by pharmacological inhibitors or STIM1/Orai1 silencing resulted in the decreased phosphorylation of the cyclin-dependent kinase CDK2 and increased expression of cyclin E, leading to the cell cycle arrest in G1/S transition accompanied with autophagy [185]. Therefore, these studies established the role of SOCE mediated by the STIM1 and Orai1 as the molecular determinants responsible for the Ca^2+^ fluxes controlling the G1/S cell cycle checkpoint of cervical cancer cells [185]. Regarding the role of STIM2 in cervical cancer cell proliferation, results from the individual or simultaneous silencing of STIM1/STIM2 suggested that both STIM1 and STIM2 contribute to the cell proliferation [162], at least partly through the regulation of SOCE during G1/S transition [185]. Furthermore, the growth of human cervical cancer xenograft in the SCID mice was attenuated by the interference with STIM1 expression or blockade of SOCE activity, demonstrating the in vivo significance of SOCE in cell proliferation [184]. These studies highlight the important roles of the STIM-mediated SOCE pathway in controlling cervical cancer cell proliferation via the regulation of the G1/S cell cycle checkpoint.

#### 4.1.2. Tumor Angiogenesis

Tumor angiogenesis is the process of the recruitment of a new blood vessel network by which the uncontrolled growth, expansion, and dissemination of cancer cells are sustained with the supportive microenvironment enriched in oxygen and various nutrients [186]. The functional significance of STIM1-dependent SOCE in tumor angiogenesis supporting the progression of cervical cancer was revealed from the study using the model of SiHa cervical cancer cells [184]. Results from the mouse tumor xenograft model of cervical cancer showed that STIM1 silencing or SOCE blockade resulted in a reduction in tumor neovascularization and tumor growth. Measurement of the secretions of vascular endothelial growth factor (VEGF), a potent inducer of vascular endothelial cell proliferation and migration, showed that STIM1 expression regulated VEGF-A productions from cervical cancer cells. Together with other investigations dissecting the functional roles of SOCE in vascular endothelial cells [187,188,189], it is suggested that STIM1-mediated Ca^2+^ machinery can be an attractive therapeutic target for strategies against tumor neovascularization.

#### 4.1.3. Cell Migration

It has been well established that SOCE dependent Ca^2+^ signaling network plays a vital role in the cellular migration of both non-cancerous and cancer cells through orchestrating cytoskeletal reorganization, focal adhesions, and direct sensing [190,191]. Results from STIM1 overexpression or silencing, as well as the pharmacological blockade of SOCE in cervical cancer, showed that STIM1-mediated SOCE is crucial for the migratory capability of cervical cancer cells [163,184,192]. The molecular mechanisms by which STIM1-dependent SOCE regulates cervical cancer cell migration mainly are through the Ca^2+^-dependent molecules controlling the focal adhesion turnover and actomyosin contractility, including calpain protease, myosin light chain kinase (MLCK), and focal adhesion proteins protein-rich tyrosine kinase (Pyk2), focal adhesion kinase (FAK), and talin. Therefore, it is proposed that STIM1-mediated Ca^2+^ influx regulates the contraction of myosin II-based actomyosin via the phosphorylation of the myosin II regulatory light chain by the Ca^2+^-dependent MLCK [192]. Moreover, the recruitment of the active focal adhesion proteins to nascent cell adhesions at the cell front, as well as the activation of the Ca^2+^-sensitive protease calpain at the rear end, are dependent on STIM1 expression or activity. Therefore, by altering the focal adhesion turnover and actomyosin contractility of cancer cells, STIM1-dependent SOCE promotes tumorigenesis and tumor metastasis of cervical cancers.

### 4.2. Diagnostic and Prognostic Values of SOCE in Cervical Carcinogenesis

Aberrated overexpression of STIM1 or Orai1 and thus upregulated SOCE activity have been observed in several types of human cancers, including cervical cancers. STIM1 and Orai1 are overexpressed in tumor tissues when compared with non-cancerous or precancerous tissues in patients with cervical cancers [162,163,184,185]. The distinct distribution of overexpressed STIM1 was identified in the invasive tumor front of the surgical specimens of human cervical cancer [193]. The studies in human cervical cancer indicated that poorer clinical outcomes, such as larger tumor size and elevated lymph node metastasis, are correlated with STIM1 upregulation in primary tumors [184], highlighting the clinical significance of STIM1 in cervical cancer progression.

Regarding STIM2, our recent study on a limited number of surgical specimens of cervical cancer showed a decreased tumoral STIM2 expression when compared with non-cancerous epithelium, whereas a higher tumoral STIM2 level when compared with invasive tumor front [162]. The simultaneous STIM1 and STIM2 immunostaining showed that, despite the overexpression of both isoforms in tumor tissues, STIM1 is the principle ER Ca^2+^-sensing molecule detected in the invasive tumor front [162]. These imply that STIM1 is associated with tumor growth and invasion, whereas STIM2 is mainly correlated with tumor growth. Therefore, using the STIM1/STIM2 ratio as a marker of cervical cancer aggressiveness might be promising and worth further evaluation.

### 4.3. Recent Development of Therapeutics Targeting SOCE in Cervical Carcinogenesis

Given the importance of SOCE tumor biology and cancer progression, it is plausible to suggest that the blockade of STIM1/Orai1-dependent Ca^2+^ signaling can be a practical therapeutic approach for cervical cancer. Studies on preclinical animal models have demonstrated the potentials of several small-molecule SOCE inhibitors in cancer therapies [194,195,196,197,198]. However, these SOCE inhibitors have not been approved for clinical use for cancer therapies. For example, SKF-96365 and 2-aminoethoxydiphenyl borate (2-APB), two of the potent pharmacological blockers of SOCE, prevented the tumor growth and angiogenesis in human cervical cancer-implanted SCID mice [184]. Further evidence from the overexpression or silencing of STIM1 and Orai1 supported that in vivo anti-tumor effects of SKF-96365 or 2-APB involve the blockade of STIM1/Orai1 complex [163,184].

Due to the ubiquitous expression of STIM and Orai protein, as well as their essential roles in the human immune system, including antitumor immunity, developing cancer cell-specific SOCE modulators is essential for effective antitumor therapeutics. For example, a study in the model of human cervical cancer has suggested that the different regulatory effects on the microtubule-dependent STIM1 trafficking between non-cancerous epithelial and cancerous cells could be the key to target cancer cell-specific mechanisms of SOCE activation [163]. Reversible acetylation of α-tubulin on Lys40 is important for regulating microtubule stability and function and thus modulating cell motility [199,200,201]. The histone deacetylase 6 (HDAC6) is a unique cytosol-localized HDAC member known as a prominent α-tubulin deacetylase [202,203]. It was found that the microtubule-dependent STIM1 translocation and subsequent SOCE activation of cervical cancer cells, but not in non-cancerous epithelial cells, was abrogated upon hyperacetylation of α-tubulin by pharmacological blockade or silencing of HDAC6 [163]. Thus, the microtubule-associated HDAC6 can be a cancer-specific target of malignant phenotypes mediated by STIM1-dependent SOCE, at least for cervical cancers with upregulation of HDAC6 and STIM1.

A recent investigation demonstrated the important role of the lysosomal cysteine protease cathepsin S in regulating STIM1 trafficking [204]. It highlighted the potential of the α-ketoamide-based highly selective cathepsin S inhibitor RJW-58 in the suppression of cervical cancer cell migration and invasion of cervical cancer cells [204]. Cathepsin S, a lysosomal cysteine protease, has been reported to be associated with the degradation of the extracellular matrix, thus promoting cell migration and invasion [205]. Results of immunoprecipitation assays demonstrated that cathepsin S interacted with STIM1, which was reversed by RNAi-mediated silencing and enzymatic inhibition of cathepsin S. Analyses of confocal microscopic and super-resolution imaging indicated that cathepsin S inhibition led to STIM1 puncta accumulation in the ER and interrupted the STIM1-EB1 interaction, a critical step for STIM1 trafficking towards the cell periphery. In addition, RNAi-mediated silencing and enzymatic inhibition of cathepsin S significantly decreased SOCE and reduced the activity of downstream Ca^2+^-dependent effectors NFAT1 and Rac1. These results provide new insight into the potential of a highly-selective cathepsin S inhibitor RJW-58 as a promising anti-cancer treatment that targets microtubule-dependent STIM1 translocation and subsequent SOCE activation [204].

**Figure 3:**
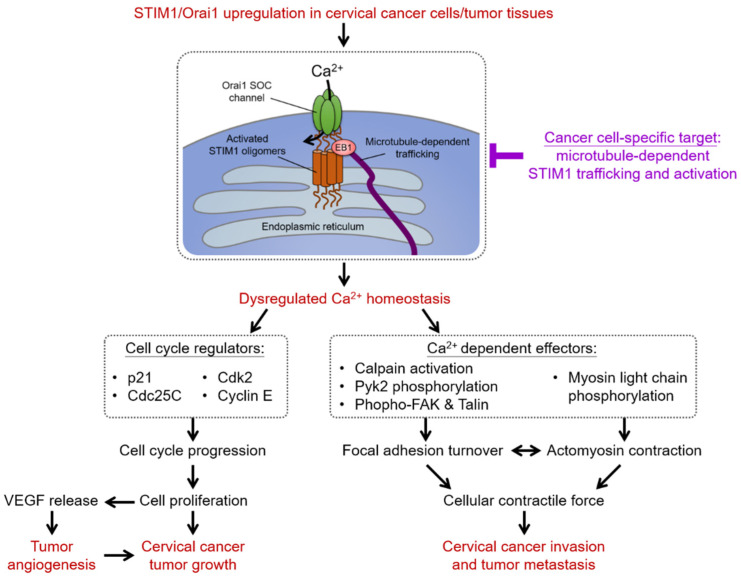
STIM1/Orai1-mediated of SOCE in cervical carcinogenesis and therapeutic implications. Ca^2+^ homeostasis is remodeled in cervical cancer cells, with Ca^2+^ influx increasing through STIM1/Orai1upregulation. STIM1-mediated SOCE controls the G1/S cell cycle checkpoint by regulating several cell cycle regulators. STIM1-mediated Ca^2+^ influx is important for the release of vascular endothelial growth factor (VEGF) from cervical cancer cells, thereby promoting tumor angiogenesis. Additionally, STIM1/Orai1-dependent Ca^2+^ signaling integrates the dynamic interactions between focal adhesion turnover and actomyosin contraction to mediate cellular contractile force and thus cell migration. Therefore, STIM1/Orai1-remodeled Ca^2+^ homeostasis is important for aggravating tumor development in vivo. Moreover, the microtubule-dependent STIM1 trafficking that is specifically modulated in cancer cells can be a cancer-specific target of malignant phenotypes mediated by STIM1-dependent SOCE, at least for cervical cancers.

The mechanisms by which STIM-mediated SOCE regulate the tumorigenesis of cervical cancer cells through regulating cell cycle progression, migration, and angiogenesis, as well as their therapeutic implications, are summarized in Figure 3.

## 5. Conclusions and Prospects

Accumulating evidence has advanced our understanding of the potentially pivotal role of membrane ion transport systems, especially those involved in cell volume regulation and intracellular Ca^2+^ homeostasis, in a variety of malignant characteristics and progression of human cervical cancer. This knowledge will stimulate the discovery of potent and selective pharmacological interventions for human cancers. The recent discovery and characterization of cryo-EM structures and activating dephosphorylation mechanism of KCC has revealed the druggable niche of KCC function to therapeutically modulate ionic fluxes and cell volume regulation in human cancer. Further investigations elucidating their structure-activity relationship will lead to the discovery and development of innovative, selective, and safe therapeutics for patients with cancer. Regarding the STIM1/Orai1-mediated SOCE, further studies aiming at developing potent and selective inhibitors that target cancer cell-specific microtubule-dependent STIM1 trafficking mechanism on SOCE activation will facilitate better therapeutic approaches.

## Data Availability

This review article did not report any data.
